# Supplementary figures and images for: RAB3 phosphorylation by pathogenic LRRK2 impairs trafficking of synaptic vesicle precursors
Source: J Cell Biol. 2024 Mar 21;223(6):e202307092. doi: 10.1083/jcb.202307092 (PMC10959120; doi:10.1083/jcb.202307092)

# Source Data (Figure 4)

E

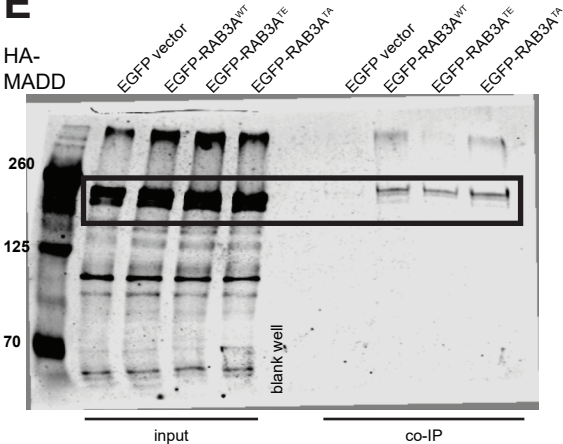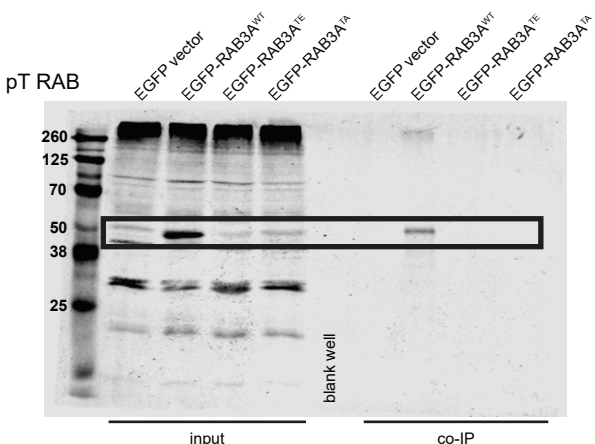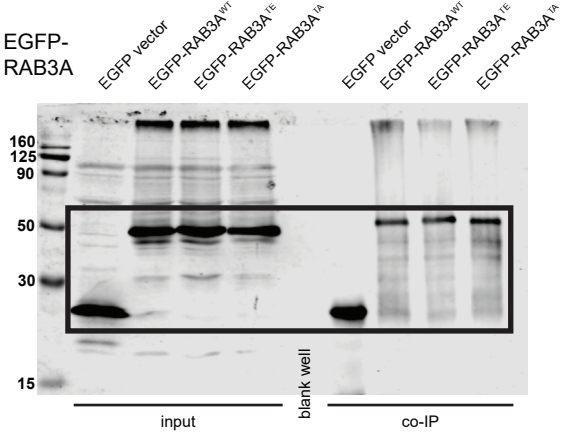

G

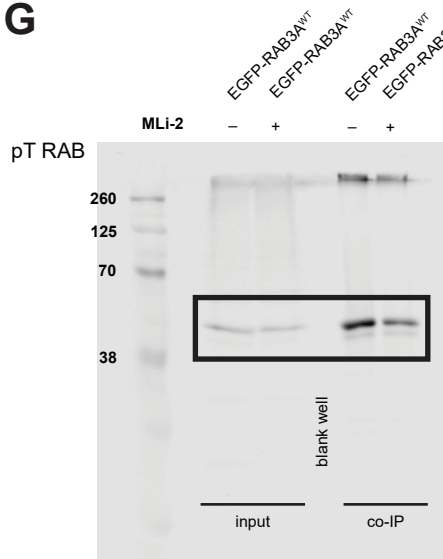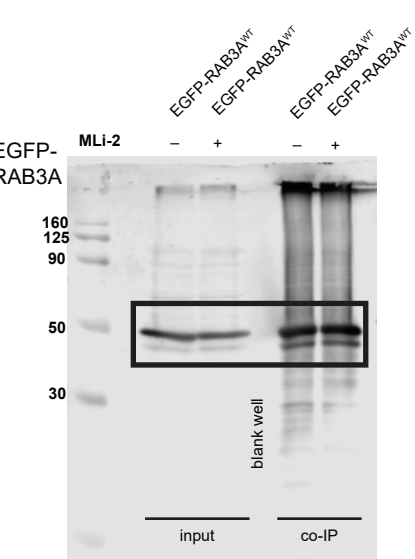

I

HA-MADD

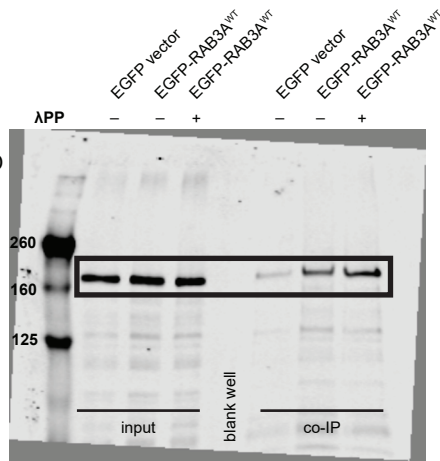

pT RAB

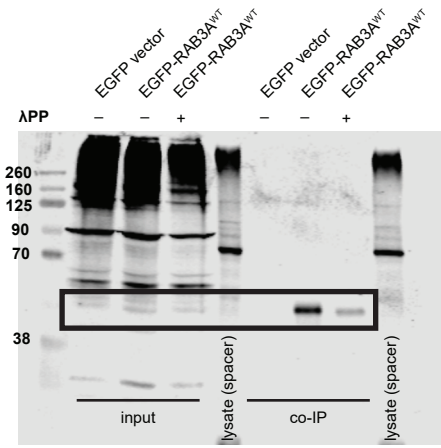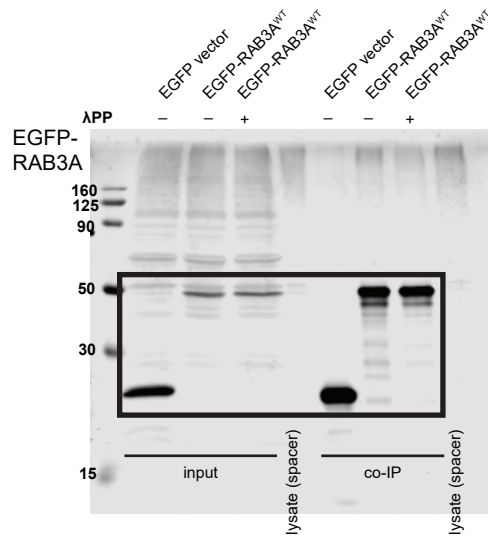

Supplement: SourceData F4 — is the source file for Fig. 4. [file JCB_202307092_SourceDataF4.pdf]

# Source Data (Figure 5)

**A**

RAB-GDI1  
(endogenous)

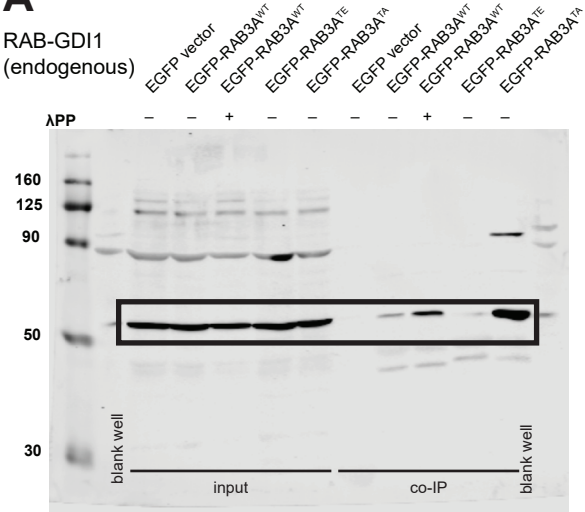

RAB3GAP2  
(endogenous)

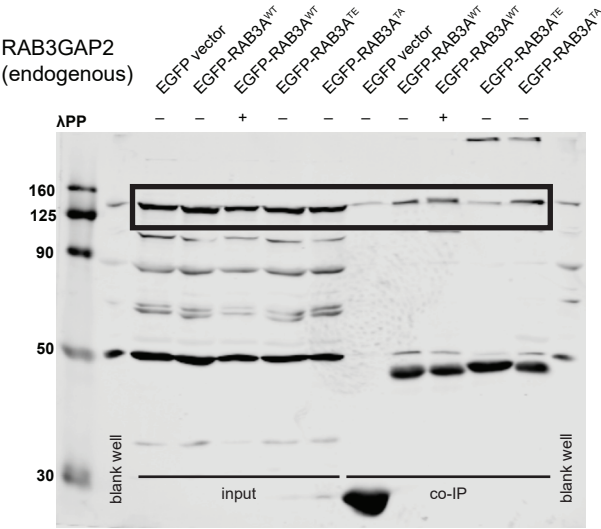

pT RAB

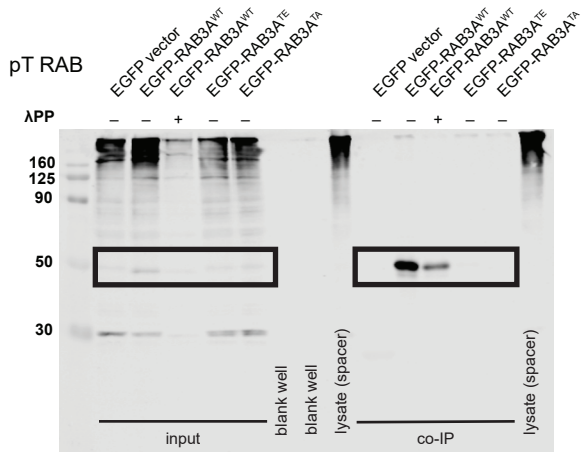

EGFP-RAB3A

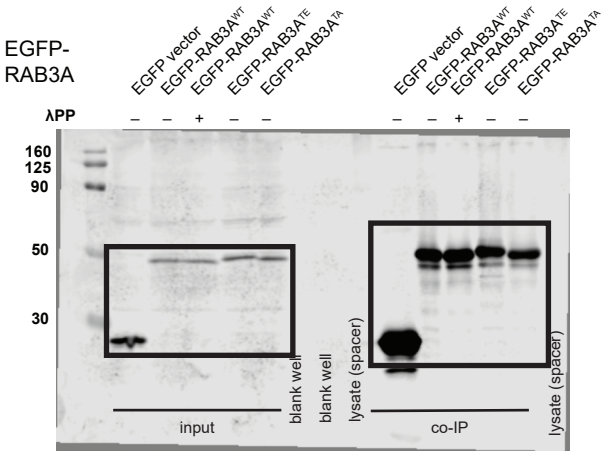

Supplement: SourceData F5 — is the source file for Fig. 5. [file JCB_202307092_SourceDataF5.pdf]

# Source Data (Figure S2)

**B**

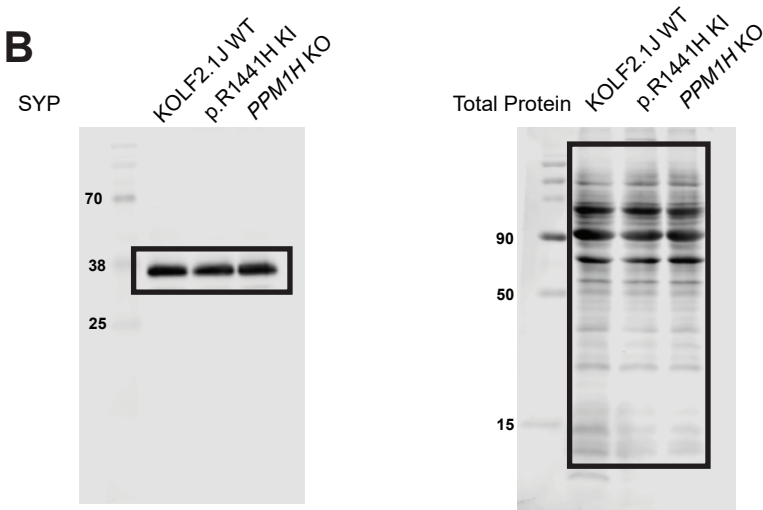

**C**

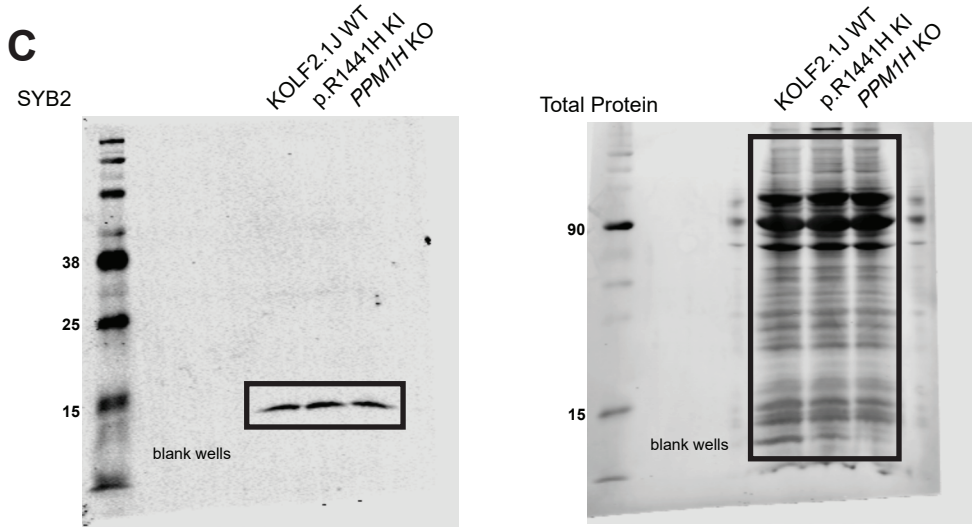

**D**

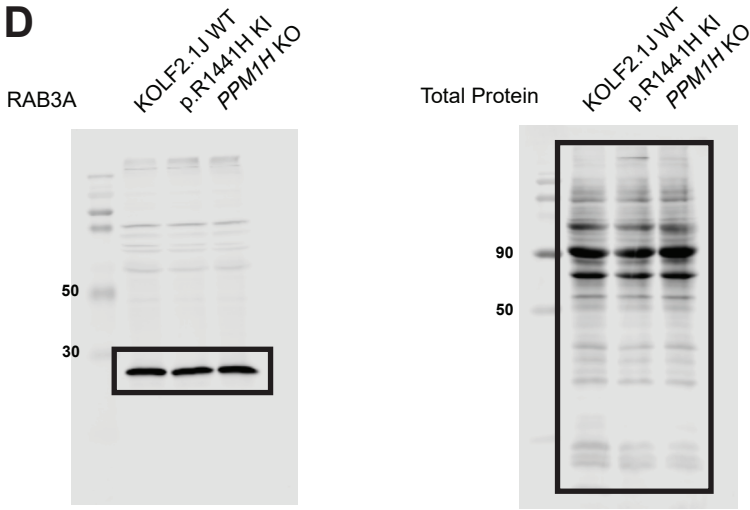

Supplement: SourceData FS2 — is the source file for Fig. S2. [file JCB_202307092_SourceDataFS2.pdf]

# Source Data (Figure S3)

HA-  
MADD

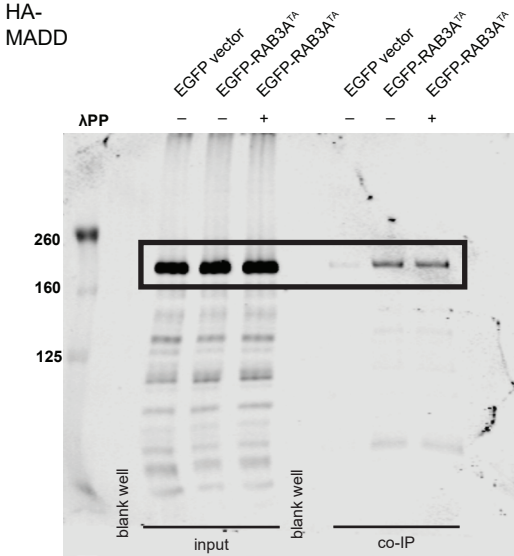

EGFP-  
RAB3A

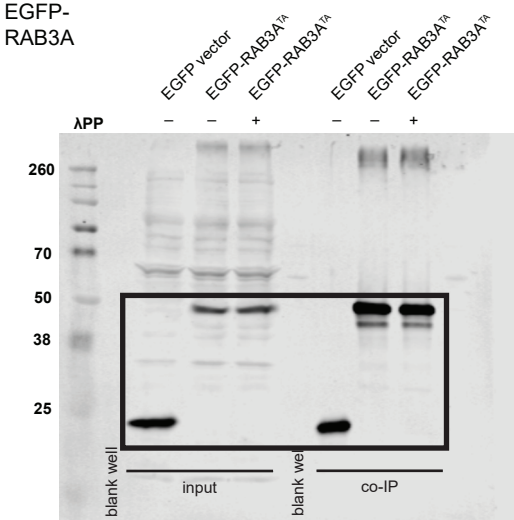

Supplement: SourceData FS3 — is the source file for Fig. S3. [file JCB_202307092_SourceDataFS3.pdf]

# Source Data (Figure S4)

A

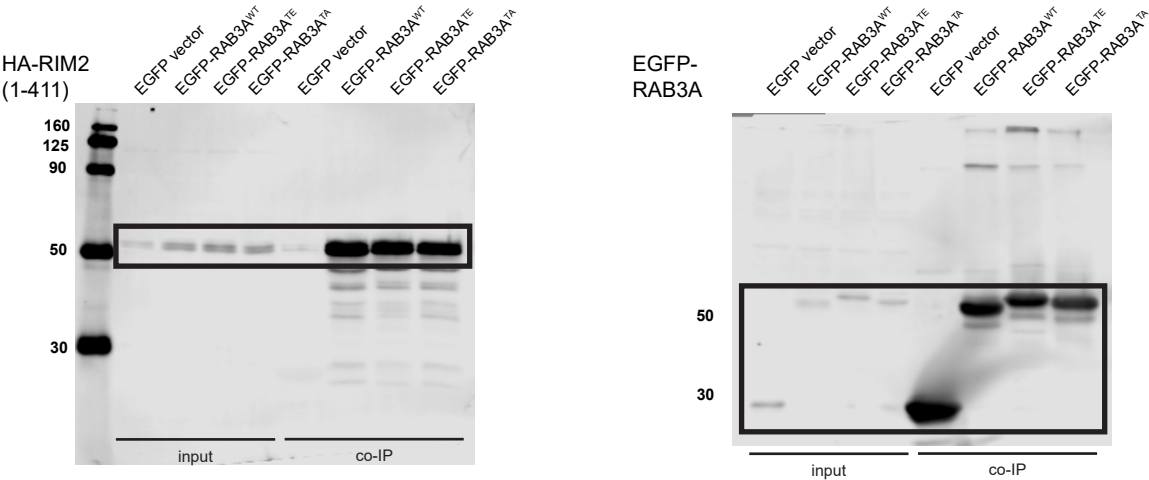

B

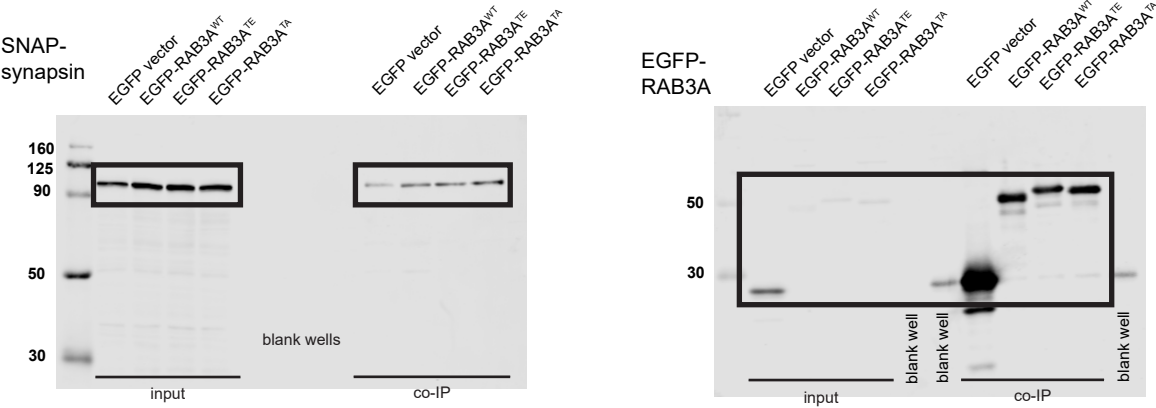

Supplement: SourceData FS4 — is the source file for Fig. S4. [file JCB_202307092_SourceDataFS4.pdf]
